# Impact of Removing Nonprescription Codeine in Australia: Protocol for a Prospective Cohort Study

**DOI:** 10.2196/15540

**Published:** 2020-03-13

**Authors:** Jacqui McCoy, Suzanne Nielsen, Raimondo Bruno

**Affiliations:** 1 University of Tasmania Hobart Australia; 2 Monash University Melbourne Australia

**Keywords:** codeine, opioids, dependence, rescheduling, drug policy

## Abstract

**Background:**

On February 1, 2018, Australia rescheduled codeine to a prescription-only medication. Many concerns were associated with this change, including increased financial costs, reduced service accessibility, the potential for poorer pain management, and a decline in physical and mental health if codeine could not be accessed. In the research literature, there is limited knowledge about the long-term consequences of rescheduling pharmaceutical opioids and, as Australia has followed many countries in implementing a restriction on codeine, further study of these consequences is critical.

**Objective:**

The goal of this study was to examine the impact of rescheduling codeine from an over-the-counter (OTC) product to a prescription-only medicine on the primary measures of codeine use and dependence in a prospective cohort of people who are frequent consumers of OTC codeine. Secondary measures included pain and self-efficacy, health service use, and mental health.

**Methods:**

The Codeine Cohort study aimed to recruit 300 participants in Australia who regularly (at least a few times per week for the past 6 months) used OTC codeine. Using an online survey, participants were followed up at three time points (February 2018, June 2018, and February 2019) after codeine was rescheduled.

**Results:**

All four waves of data collection are complete, with the final round of data collection finalized in August 2019. Data analyses are yet to be completed. Information on demographics, codeine use and dependence, physical and mental health, medication use, and health service use will be analyzed using mixed models.

**Conclusions:**

Results of this study will provide insight into the effectiveness of regulatory restriction in curtailing nonmedical use of and harms associated with codeine. Additionally, results will explore positive and negative outcomes of codeine rescheduling for individual patients, which informs health professionals who support patients who use codeine and further community education.

**International Registered Report Identifier (IRRID):**

DERR1-10.2196/15540

## Introduction

### Background

Codeine is the most commonly used opioid in the world [1]. Regulation of its availability varies among countries; in New Zealand, the United Kingdom, most of Canada, and Ireland, codeine is available as an over-the-counter (OTC) preparation and is often combined with paracetamol or a nonsteroidal anti-inflammatory drug (NSAID) such as ibuprofen [2]. Despite its wide use, there are a number of concerns about codeine as an analgesic, with risks of prolonged misuse of OTC codeine-ibuprofen products including life-threatening complications such as gastric bleeds, renal failure, hypokalemia, and opioid dependence [3,4].

In addition to risk of serious harm, there is limited evidence for the addition of low-dose codeine (16 to 25 mg of codeine per dose) to paracetamol or ibuprofen preparations for improved pain relief [5-9]. This, coupled with the known availability of effective nonopioid alternatives for pain relief [10-12], raises concerns about the place of low-dose codeine in ongoing pain management.

International awareness of the misuse of OTC pharmaceuticals containing codeine is growing [13-15], and government responses have predominantly focused on upscheduling (increasing prescribing restrictions), improving guidelines and procedures around the supply of low-dose codeine preparations. For instance, the United States, Japan, India, and most of Europe are moving toward making pharmaceuticals containing codeine prescription-only medications [16,17].

Although upscheduling is an important regulatory response, this is not reflected with many studies in the research literature. For example, when hydrocodone combination products became restricted in the United States in 2014 [18], few studies explored the impact of this change on individuals. One study was conducted through a pain association in the United States, following thousands of individuals seeking support [19]. An online survey was used to evaluate the short-term (100 days) impact following the rescheduling of hydrocodone. Participants reported being placed on less effective medications, increased costs, inconvenience, and negative shifts in their relationships with health professionals. As other countries consider making similar changes to codeine and other opioids, further research to review the longer term implications of rescheduling is needed. Unintended potential consequences of rescheduling and restricting supply include fewer pain relief options for consumers, movement toward stronger opioids, and an increased burden on health care systems [20,21].

Restriction of OTC codeine product availability in Australia began in May 2010 [22]. Codeine preparations were required to be stored behind the counter and pack sizes reduced to a maximum 5-day supply. Despite these restrictions, OTC codeine remained widely used. In 2013, more than 15 million packets of OTC codeine were sold in Australia [23], representing almost one pack per person aged over 15 years [24]. Furthermore, OTC codeine accounted for 37% of opioid sales in the general community [24]. Despite the initial upscheduling, concerns in Australia with the misuse of OTC codeine products increased [25], with reports of growing numbers of patients with codeine dependence presenting to emergency departments and drug treatment services [4,26,27].

In December 2016, the Therapeutic Goods Administration of Australia determined that the limited therapeutic gain offered was outweighed by the evidence of harm associated with OTC codeine use, and the products were moved to schedule 4 (prescription only), effective February 1, 2018 [28]. This decision was based on concerns regarding the harmful side effects of codeine use as well as the known availability of safer OTC products (eg, ibuprofen-paracetamol combinations) with comparable efficacy [28].

Prior to the upscheduling of codeine in Australia, regular consumers of OTC codeine and health professionals queried whether this decision would, in fact, reduce codeine-related harms [29]. Participants were concerned about other unintended consequences, including poorer pain management, limited physical health, and increased emotional distress. It is important to study the outcomes of rescheduling decisions to determine if health professional and consumer concerns are realized and also to understand the impact of rescheduling decisions more broadly, given that this is a common regulatory lever used by governments internationally. To address this need, this study sought to evaluate the impact of rescheduling codeine in a prospective cohort of regular codeine users who were assessed 3 months prior to and followed for 12 months after the February 2018 rescheduling.

### Objectives

The goal of this study was to examine the impact of rescheduling codeine from an OTC product to a prescription-only medicine on the primary measures of codeine use and dependence in a prospective cohort of people who are frequent consumers of OTC codeine. Secondary measures included pain and pain self-efficacy, health service use, and mental health.

## Methods

### Study Design and Setting

The Codeine Cohort study was an online-based single-center prospective longitudinal study. Participants were recruited in November 2017, and those who were eligible were invited to complete the first online survey for November 2017 (baseline). Codeine was rescheduled to prescription-only on February 1, 2018, and follow-up surveys were completed 1 month (end of February 2018), 4 months (June 2018), and 12 months (February 2019) after this rescheduling. These time points were selected to allow sufficient evaluation of the immediate, short-term, and long-term effects of the rescheduling. To allow as many participants as possible to respond, data collection for the third time point (February 2019) was finalized by August 2019. Data analyses are yet to be completed.

### Ethics Approval and Consent to Participate

This study was approved by the Human Research Ethics Committee of the University of Tasmania (HREC reference number: H0016685).

### Participants

Participants were required to be at least aged 18 years and living in Australia. They were asked about the frequency of their codeine use at the screening stage and offered the following response options: every day, a few times a week, once a week, a few times a month, at least monthly, or less than monthly. Eligible participants were required to have used OTC codeine at least a few times per week or more for the previous 6 months. This threshold for frequency of use was based on results from a previous online study of codeine consumers where the top third of participants were using OTC codeine once a week or more [30]. A threshold of a few times per week or more was adopted to allow a sufficiently high baseline of codeine use from which any changes in this measure over time could be detected.

Participants who self-reported that they were in current treatment for codeine dependence were excluded from the study as changes in their codeine use as a result of treatment rather than policy change may confound the interpretability of the study results.

### Study Measures

Measures used in this study covered a range of domains including demographic information, health service use, pain and coping, physical and mental health, and codeine use and codeine dependence. Areas evaluated in this study were based on key concerns raised by health professionals and consumers in a previous study evaluating attitudes about codeine rescheduling in Australia [29]. Measures used were based on recommendations made by the Initiative on Methods, Measurement, and Pain Assessment in Clinical Trials [31] and previous studies exploring opiate use using online surveys [30,32]. Measures, domains, and time points at which data were collected are summarized in Table 1.

**Table 1:** Domains, measures, tools, and time points for data collection for the Codeine Cohort study.

Domain		Measure	Baseline	T1^a^	T2^b^	T3^c^
**Demographics**						
	Age, sex, accommodation	—	x			
	Education, employment	—		x		
**Health service use**						
	Use of physician, pharmacist, and emergency department for codeine	—	x	x	x	x
**Pain**						
	Pain and coping	PSEQ^d^	x	x	x	x
	Physical functioning	PEG^e^	x	x	x	x
**Mental health**						
	Depression	PHQ- 9^f^	x	x	x	x
	Anxiety	GAD-7^g^	x	x	x	x
	Codeine use and dependence	AUDADIS-5 CIDI^h^: substance abuse module	x			x
		SDS^i^	x	x	x	x
		CDS^j^	x	x	x	x
**Treatment**						
	Current medication	Self-complete 7-day medication diary	x	x	x	x
	Nonmedication treatment options	—	x	x	x	x

^a^T1: 1 month after rescheduling.

^b^T2: 4 months after rescheduling.

^c^T3: 12 months after rescheduling.

^d^PSEQ: Pain Self-Efficacy Questionnaire.

^e^PEG: Pain Intensity, Enjoyment of Life and General Activity Assessment Tool.

^f^PHQ-9: Patient Health Questionnaire, 9-item.

^g^GAD-7: Generalized Anxiety Disorder 7-item Scale.

^h^AUDADIS-5 CIDI: Alcohol Use Disorder and Associated Disabilities Interview Schedule–5 Composite International Diagnostic Interview.

^i^SDS: Severity of Dependence Scale.

^j^CDS: Codeine Dependence Scale.

### Measures

#### Primary Measures

##### Alcohol Use Disorder and Associated Disabilities Interview Schedule–5

Past year codeine use disorder symptoms were assessed using the Alcohol Use Disorder and Associated Disabilities Interview Schedule–5 (AUDADIS-5) [33]. The reliability and validity of the AUDADIS-5 in relation to substance abuse and dependence disorders for a range of drugs is well documented in several international studies [34,35]. To assess withdrawal symptoms, 12 symptoms were taken from the Composite International Diagnostic Interview (CIDI): substance abuse module. The CIDI is a standardized diagnostic interview designed for assessing mental disorders (including substance use disorders) according to the *International Statistical Classification of Diseases and Related Health Problems, Tenth Revision,* and *Diagnostic and Statistical Manual of Mental Disorders, Fifth Edition* (DSM-5) [36]. Diagnosis of codeine withdrawal was operationalized consistently with DSM-5 opioid withdrawal criteria [37] (at least 3 opioid withdrawal symptoms present concurrently at a distressing level or withdrawal relief). As the AUDADIS-5 may only be administered every 12 months, this measure was used at baseline (November 2017) and the third time point (February 2019).

##### Medication Diary

A medication diary was used to assess all medication use retrospectively for the past 7 days. Included were questions about the medication name, strength (mg), dose, number of times taken per day, and how many days that dose was taken across the last week.

#### Secondary Measures

##### Severity of Dependence Scale

The Severity of Dependence Scale (SDS) was used as a brief screener for possible codeine dependence. This has been validated with a range of substances, including heroin, cocaine, amphetamines [38-40], benzodiazepines [41], cocaine [42], cannabis [43], and alcohol [44]. In addition to a range of substances, the SDS has also been validated with problematic analgesic use (including combination products containing codeine) [45] where a cutoff of 5 or more demonstrated reasonable sensitivity (72.3%) and specificity (78.6%) for identifying individuals who may be problematic users of analgesics [45,46].

##### Codeine Dependence Scale

The Codeine Dependence Scale (CDS) was used as an additional measure of possible codeine dependence [47]. The CDS has 4 questions and is statistically validated against the SDS (based on an SDS score of ≥5). It has a cutoff value of ≥2 and has high sensitivity (84%) and specificity (94%) for identifying likely cases of codeine dependence [47].

##### Pain Intensity, Enjoyment of Life, and General Activity Assessment Tool

The Pain Intensity, Enjoyment of Life, and General Activity Assessment Tool (PEG) is a brief measure of pain with 3 items evaluating pain intensity and the level of interference in general activity and enjoyment of life [48]. It is derived from the widely used Brief Pain Inventory. The PEG demonstrates excellent internal consistency and good construct validity, with a sensitivity to change (at 6 months postbaseline) consistent with the Brief Pain Inventory [48] and demonstrated responsiveness to clinical interventions [49].

##### Pain Self-Efficacy Questionnaire

The Pain Self-Efficacy Questionnaire (PSEQ) is a 10-item questionnaire that assesses the confidence people feel in completing a number of activities despite experiencing pain [50]. The PSEQ has demonstrated adequate reliability and validity [51] and has been used in a wide variety of clinical populations, countries, and languages [52-58].

##### Patient Health Questionnaire–9 Item

The Patient Health Questionnaire–9 item (PHQ-9) is a 9-item questionnaire that examines symptoms of depression as defined in the DSM-5 [59,60]. Scores indicate the severity of depressive symptoms, with a maximum score of 27. Scores of 5, 10, 15, and 20 represent mild, moderate, moderately severe, and severe depression, respectively [60]. The PHQ-9 has demonstrated validity [61,62] and has been used widely in research, clinical practice, and surveys of mental health [63-68].

##### Generalized Anxiety Disorder 7-Item Scale

The Generalized Anxiety Disorder 7-item Scale (GAD-7) is a 7-item questionnaire that evaluates symptoms of generalized anxiety disorder. Scores of 5, 10, and 15 indicated mild, moderate, and severe anxiety, respectively [69]. The GAD-7 has demonstrated satisfactory validity and strong clinical utility in primary care settings and in the broader population [69-71].

##### Health Service Use

The health service use questions included in this online survey assessed the number of visits with a physician, pharmacist, and emergency department in the past 3 months related to codeine use. These questions were adapted from the Pain and Opioids In Treatment cohort study [72].

### Participant Recruitment and Procedure

Participants were recruited through professional and personal networks; posts on relevant internet health forums and organizations (eg, Pain Australia, Pharmacy Guild); and University of Tasmania, University of New South Wales, and National Drug and Alcohol Research Centre media releases and emails to research participants from a previous study who had consented to be contacted for future participation opportunities [29]. Social media, including Facebook advertisements and Twitter, was also used, as it has been demonstrated to enable recruitment of greater populations of participants with high levels of substance use and associated issues [73].

Potential participants were directed to an internet survey using Research Electronic Data Capture [74], where they were given detailed study information and asked to provide informed consent and answer a few brief screening questions to assess eligibility (including questions on age, living location, and frequency of codeine use). Eligible participants were invited to complete the first online survey (November 2017, baseline) by email using a unique and secure link. The first survey took between 20 and 30 minutes to complete. Participants were placed in a prize draw to win one of twenty $100 gift vouchers at baseline (November 2017) and were reimbursed with a $20 gift voucher at each of the three follow-up time points (February 2018, June 2018, and February 2019). Data collection for the final time point was completed by August 2019. Contact details including an email address and phone number were collected at baseline to enable communication over the course of the study.

A number of cohort management strategies were employed based on a Cochrane review and meta-analysis of participant retention [75]. First, participants were reimbursed for their research contribution. Second, a number of methods were used to contact participants and encourage them to participate, including reminder emails when a follow-up stage of data collection had commenced. Additionally, text messages with their unique survey link and phone calls to participants were made, if email reminders were insufficient in encouraging participants to complete the survey at that time point.

Eligibility criteria were not disclosed so that participant responses could not be tailored to ensure study entry. Part of a participant’s eligibility was determined from their answers to a screening question about the frequency of their codeine use in the last 6 months. Finally, at baseline (November 2017) participant reimbursement was a prize draw rather than a voucher to reduce the likelihood of those who would participate for monetary gain.

### Data Analysis

Data analyses for the Codeine Cohort study are yet to be completed. Assuming statistical assumptions are met for the primary outcome of total daily codeine dose (mg; continuous variable), mixed-model analyses will be conducted. For the primary outcome of codeine dependence (categorical), generalized linear mixed models will be applied. In the case of missing data for both aims, sensitivity analyses will be conducted with a full information dataset (all data collected from each individual), a last observation carry-forward dataset, and a dataset derived from multivariate imputation of missing data. In the event of the number of usable observations not meeting requirements from power calculations, regression results from the dataset with multivariate imputation will be used (subject to sensitivity analyses).

Logical model-building processes (ie, stepwise regression) will be applied to the development of these models with covariates including demographics, physical and mental health, and pain. Secondary analyses will be conducted using similar analytic frameworks taking into account the nature of the outcome variable under study.

### Sample Size and Power

Power analyses were conducted to estimate a sufficient sample size required to assess the potential effects of codeine restriction on two primary outcomes: continuous measures of codeine use per day and rates of dependence (categorical).

### Average Total Codeine Use per Day (Averaged Dose in Milligrams Over the Preceding 7 Days)

Using a repeated measures analysis of variance framework with four assessment points and 200 participants, there is power of 0.8 or greater to detect an effect size as small as Cohen *f*=0.1 as statistically significant for the main effect of time where there are correlations among repeated measurements as low as *r*=.20 and even smaller magnitude effects (*f*=0.08) where correlations are the more likely *r*=.50. In a Web survey of codeine consumers by Nielsen et al [76], daily codeine use had a mean of 68 (SD 72) mg. These power calculations suggest that there would be sufficient power to identify a drop from 68 to 53 mg per day in the daily codeine dose variable as statistically significant, assuming this range of correlations and this standard deviation of scores. This is a little more than a single 12.8 mg nonprescription (OTC) codeine tablet, and therefore, is of an appropriate magnitude for this study.

### Rates of Dependence

Detailed assessment of dependence was completed at baseline and T3 (only) using the AUDADIS-5 [33]. In order to compare rates of dependence at these two time points, a comparison of correlated proportions can be made with a McNemar test. A sample size of 249 pairs achieves 80% power to detect a difference between two paired proportions of 0.1 at an alpha level of .05, when the proportion at baseline is 0.2 and the proportion at T3 is 0.1. The proportion of discordant pairs is 0.3. Approximately 20% of regular OTC codeine users are dependent (46); therefore, it was assumed that 20% of the study cohort could be defined as dependent at baseline. Assuming a 20% dropout rate [77], a sample size of 300 was considered appropriate to meet both of these aims.

## Results

All four rounds of data collection for the Codeine Cohort study are complete. Data analyses are underway currently and results from the study will be published in 2020.

## Discussion

### Preliminary Findings

Prior to the rescheduling of OTC codeine in Australia, individuals who regularly used codeine (consumers) indicated their concern whether rescheduling would minimize codeine-related harms (including dependence) and the impact the requirement of a prescription for codeine would have on their emotional and physical health, their pain management, and overall quality of life (29). Pharmacists shared consumer concerns and were focused on the burden regular doctor appointments would create in terms of finances for consumers.

Examination of administrative data such as sales, prescriptions, and emergency presentations provides some information as to the success of upscheduling at the population level. This study will contribute to an improved understanding of the outcomes, positive and negative, associated with codeine rescheduling for the individual patient, which informs where further community education and intervention are needed most. Also, by exploring whether codeine rescheduling leads to reduced codeine use and levels of dependence, this study will provide insight into the overall effectiveness of regulatory restriction in curtailing misuse of pharmaceutical opioids.

### Strengths and Limitations

In terms of strengths, this is a novel study; to the knowledge of the authors, a prospective online cohort investigating the longer term impacts (over 12 months) of codeine rescheduling on the individual patient has not been studied before. Additionally, a number of measures were used, including multiple measures of codeine dependence. In terms of limitations, the study has a modest sample size and the generalizability of findings to the general population might be limited, as the sample comprised self-selected participants in an online study.

## Abbreviations

AUDADIS-5: Alcohol Use Disorder and Associated Disabilities Interview Schedule–5
CIDI: Composite International Diagnostic Interview
CDS: Codeine Dependence Scale
DSM-5: Diagnostic and Statistical Manual of Mental Disorders, Fifth Edition
GAD-7: Generalized Anxiety Disorder 7-item Scale
NSAID: nonsteroidal anti-inflammatory drug
OTC: over-the-counter
PEG: Pain Intensity, Enjoyment of Life, and General Activity Assessment Tool
PHQ-9: Patient Health Questionnaire–9
PSEQ: Pain Self-Efficacy Questionnaire
SDS: Severity of Dependence Scale
